# ASPP2 enhances Oxaliplatin (L-OHP)-induced colorectal cancer cell apoptosis in a p53-independent manner by inhibiting cell autophagy

**DOI:** 10.1111/jcmm.12435

**Published:** 2014-12-23

**Authors:** Ying Shi, Yue Han, Fang Xie, Anna Wang, Xiaokun Feng, Ning Li, Hongliang Guo, Dexi Chen

**Affiliations:** a Beijing Youan Hospital, Capital University of Medical SciencesBeijing, China; b Beijing Institute of HepatologyBeijing, China; c Shandong Tumor Hospital, School of Medicine and Life Sciences, University of Jinan, Shandong Academy of Medical SciencesJinan, China

**Keywords:** ASPP2, colorectal cancer, apoptosis, p53, autophagy

## Abstract

Inactivation of p53-mediated cell death pathways is a central component of cancer progression. ASPP2 (apoptosis stimulated protein of p53-2) is a p53 binding protein that specially stimulates pro-apoptosis function of p53. Down-regulation of ASPP2 is observed in many human cancers and is associated with poor prognosis and metastasis. In this study, ASPP2 was found to enhance L-OHP-induced apoptosis in HCT116 p53^−/−^ cells in a p53-independent manner. Such apoptosis-promoting effect of ASPP2 was achieved by inhibiting autophagy. Further experiments with ASPP2 RNA interference and autophagy inhibitor (3-methyladenine, 3-MA) confirmed that ASPP2 enhanced HCT116 p53^−/−^ cell apoptosis *via* inhibiting the autophagy. The association of cell death and autophagy was also found in ASPP2^+/−^ mice, where colon tissue with reduced ASPP2 expression displayed more autophagy and less cell death. Finally, colorectal tumours and their adjacent normal tissues from 20 colorectal cancer patients were used to examine ASPP2 expression, p53 expression and p53 mutation, to understand their relationships with the patients' outcome. Three site mutations were found in p53 transcripts from 16 of 20 patients. ASPP2 mRNA expressions were higher, and autophagy level was lower in the adjacent normal tissues, compared with the tumour tissues, which was independent of both p53 mutation and expression level. Taken together, ASPP2 increased tumour sensitivity to chemotherapy *via* inhibiting autophagy in a p53-independent manner, which was associated with the tumour formation, suggesting that both p53 inactivation and ASPP2 expression level were involved in the sensitivity of colorectal cancer to chemotherapy.

## Introduction

p53 pathway mediates many cellular processes including apoptosis, cell cycle regulation and DNA repair 1–3. Because of its significance, p53 function is tightly regulated by multiple mechanisms. p53 is activated by a number of cellular stresses including hypoxia, ionizing radiation, DNA damage and chemotherapeutic drugs. The function of p53 can be disrupted through p53 gene mutations, alterations of upstream activating pathways, or alterations in downstream components that mediate p53 signals 4. Clearly, several factors influence the p53 pathway; however, the mechanisms by which the cellular context determines the specific p53 function, such as apoptosis *versus* cell cycle arrest, are not fully understood.

Apoptosis is independent of p53, and most colorectal cancer cells do not contain functional p53 protein. Therefore, change in tumour suppressor p53-mediated apoptosis pathway is one of the key genetic steps in colorectal carcinogenesis 5–7. However, p53 mutations generally occur late in colorectal tumourigenesis, near the transition to malignancy 8,9, suggesting that multiple apoptotic controls including p53 interaction proteins exist to control neoplasia in colorectal carcinomas.

ASPP2 is a p53 binding protein, which enhances damage-induced apoptosis at least in part through a p53-mediated pathway 10. Additional evidence suggests that p53-independent pathway may also be modulated by ASPP2. Reduced expression of ASPP2 was found to be associated with poor prognosis and metastasis in human cancers 11,12. In mouse models, ASPP2 is regarded unequivocally as a tumour suppressor that functions as an activator of the tumour suppressor function of p53 13. These findings suggest that ASPP2 proteins may play an important role in human tumour development. However, the mechanism of ASPP2 in modulating p53 dependent and independent apoptosis functions remains poorly understood.

In this study, we showed that ASPP2 solely stimulated chemotherapy-induced cell apoptosis *in vitro* in a p53-independent manner *via* inhibiting the cell autophagy. Overexpression of ASPP2 also inhibited autophagy and enhanced apoptosis in mice. Study of clinical samples showed that ASPP2 was highly expressed in normal tissues adjacent to colorectal tumours, suggesting that low levels of ASPP2 in colorectal tumours might have contributed to the tumour survival in both p53-dependent and -independent manners following chemotherapy.

## Materials and methods

### Cell culture conditions and reagents

HCT 116 p53^−/−^ human colon cancer cell line 14 was a gift from Dr. Charles Lopez (Oregon Health and Science University, Portland, Oregon). Cells were grown in Dulbecco's modified Eagle's medium (DMEM) with 10% foetal bovine serum, 290 μg l-glutamine, 100 units penicillin, and 100 μg streptomycin (Invitrogen Life Technology) per ml under 37°C and 5% CO_2_.

### Cell death assay

Flow cytometric analysis was performed with BD FACSCanto II (Becton Dickinson). HCT116 (p53^−/−^) cells were plated 24 hr prior to transfection with ASPP2-rAd or GFP-rAd at a concentration of 1 × 10^6^ PFU/ml. After treatment with L-OHP (100 μM) for 12 hr, the cells were washed twice with cold PBS and resuspended in Annexin V binding buffer (SouthernBiotech) and analysed by flow cytometry. (Here, we chose 100 μM L-OHP and 12 hr according to our preliminary experiment shown in Figure S1.)

### RNA interference

SiRNA oligos (GenePharma Co., Ltd, Shanghai, China) targeting ASPP2 were as follows: UAUGCAGAGACGUGGUGGATT (1) and UCCACCACGUCUCUGCAUATT (2). The negative control RNA oligo sequence was UUCUCCGAACGUGUCACGUTT. The siRNAs were transfected using Fugene HD (Promega).

### Immunoblotting

Cells were lysed in high salt lysis buffer (150 mM NaCl, 1% NP-40, 0.5% deoxycholate, 0.1% SDS, 50 mM Tris [pH 8.0], and 5 mM EDTA) with protease inhibitors (10 μg/ml phenylmethylsulfonyl fluoride [PMSF]). After quantification with a BCA assay (Applygen Technologies Inc., Beijing, China), 50 μg total proteins were loaded and separated on SDS-PAGE gel, transferred to PVDF membrane, blocked with 5% non-fat milk, probed with the specified primary antibody, followed by HRP-conjugated secondary antibody, and then visualized with enhanced chemiluminescence (Pierce Supersignal) and X-ray film using standard techniques.

### Quantitative RT-PCR

Total RNAs were extracted by miRNeasy Mini Kit (Qiagen) and reverse transcribed by SuperScript® III First-Strand Synthesis System (Invitrogen). Quantitative RT-PCR (qRT-PCR) was performed with the ViiA 7DX real-time PCR system (Applied Biosystems) using the Fast SYBRGreen Master Mix (Applied Biosystems). Each reaction was performed in triplicate using 0.5 μL of cDNA in a final volume of 8 μL. Relative transcript levels of target genes were normalized with GAPDH mRNA levels. The qPCR primers used in this study are listed in Table1.

**Table 1:** Primers used by real-time PCR

	Forwards primers (5′-3′)	Reverse primers (5′-3′)
ASPP2	gtgttgcagttaggctattttgagc	gtggtgtacttacctaaaatgacatac
Bax	agtggcagctgacatgt	agggccttgagcaccagt
Bcl-2	atggcgcacgctgggagaa	atggatgtacttcagcactat
Atg5	agagtaagttatttgacgtt	tcataaccttctgaaagtgct
Atg7	aagaaataatggcggcagct	acccaacatccaaggcacta
Beclin-1	agtcgctgaagacagagcgat	tcagcccccgatgctcttcacct
GAPDH	gagccacatcgctcagacac	ggtgcaggaggcattgctga

### Immunofluorescent staining

Cells were washed with cold PBS twice and fixed in 4% paraformaldehyde/PBS for 10 min. After incubation with 1% Triton X-100 in PBS for 5 min., the cells were blocked with 2% BSA and 1% goat serum/PBS for 1 hr, followed by incubation with appropriately diluted primary antibodies for 1 hr at room temperature. Then, the cells were incubated with Alexa Fluor 488- or 594-conjugated secondary antibody (Sigma-Aldrich). DAPI (4,6-diamidino-2-phenylindole) was used to stain the nuclei.

Frozen sections of fresh tissues were prepared using the standard techniques and treated as mentioned above.

### Mice

ASPP2^+/−^ BALB/c mice were kindly provided by Lopez Lab. ASPP2 overexpression mice and control mice were obtained by injecting ASPP2-rAd or GFP-rAd into ASPP2 wild-type (WT) BALB/c mice (Academy of Military Medical Sciences, China) through the tail vein for 24 hr. All the mice were treated with L-OHP through intraperitoneal injection for 48 hr. Then colon tissues from these mice were collected freshly after being anaesthetized. Western blotting and immunofluorescence were performed as mentioned above. All studies were approved by the Ethical Committee of the Youan Hospital Center, affiliated Hospital of Capital Medical University, Beijing, and all animal care complied with the applicable health guidelines.

### Patients

Twenty patients with colorectal cancer from Shandong Tumor Hospital (Shandong, China) were included in the study. Colorectal cancer tissues and adjacent normal tissues were obtained from surgical resection. All colon cancers in this study were adenocarcinoma. Their clinical information and grades are shown in Table2. This study was approved by the local ethics committee, and informed consent was obtained from each patient. All colorectal cancer cases were confirmed through review of histology by a pathologist (S.O.) blinded to other data. Their average age was 64.7 years; 11 were male, and 9 were female. Mean body mass index (BMI; calculated as weight (kg)/[height (m)]^2^) was 25.9. Tumour differentiation was categorized as well-moderate *versus* poor (>50% *versus* ≤50% gland formation).

**Table 2:** Basic clinical, pathologic characteristics of the patients in this study

Clinical, pathologic characteristic	Values
Age, mean (SD), years	64.7 (8.3)
Sex (M/F)	11/9
Body mass index, mean (SD)	25.9 (4.5)
Stage of disease (case number I/II/III/IV)	4/8/6/2
Grade of differentiation (case number well-moderate/poor)	17/3

### Mutation in p53 transcripts analysed by RT-PCR

Total RNAs were extracted from colorectal cancer tissues and para-tumour normal tissues. PCR was performed by using the following primers: S: 5′-ATGGAGGAGCCGCAGTCAGAT-3′; AS: 5′- TCAGTCTGAGTCAGGCCCTTCTG-3′. The PCR was carried out for 35 cycles at 94°C for 30 sec., 55°C for 45 sec., and 72°C for 45 sec. Then the PCR mix (5 μl) was subjected to 4% agarose gel electrophoresis at 100 V for 30 min., and PCR product were visualized by ethidium bromide staining. The p53 transcripts were analysed by bi-directional sequencing using the same PCR primers.

## Results

### ASPP2 triggers apoptosis in a p53-independent manner

**Fig 1:**
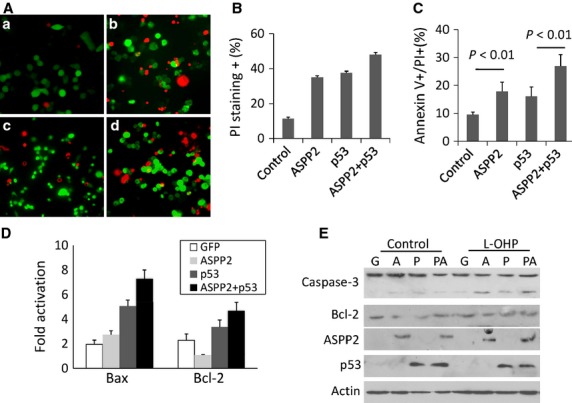
ASPP2 induced apoptosis in a p53-independent manner. (A) PI staining of HCT116 (p53^−^/^−^) cells transfected with Adenovirus followed by treatment with L-OHP; a, b, c and d were cells transfected with GFP-rAd, ASPP2-GFP-rAd, p53-GFP-rAd and ASPP2-GFP-rAd plus p53-GFP-rAd followed by treatment with L-OHP. (B) The percentage of PI-stained cells counted under a microscope. (C) The percentage of Annexin V-stained adenovirus transfected cells analysed by Flow cytometry; (D) qRT-PCR analysis of Bax and Bcl-2 mRNA in L-OHP-treated HCT116 cells, with GAPDH mRNA as internal control; (E) Immunoblot analysis of ASPP2, p53, Bcl-2 and activated caspases-3 expression in L-OHP-treated HCT116 cells. Lane 1, 2 and 3 were cells transfected with GFP-rAd, ASPP2-rAd and p53 plus ASPP2-rAd; Lane 4, 5 and 6 were cells transfected with GFP-rAd, ASPP2-rAd, p53 plus ASPP2-rAd followed by L-OHP treatment for 24 hr.

Recent study showed that ASPP2 also mediates p53-independent pathways 11,13. To identify the role of ASPP2 in cells with non-functional p53, which was confirmed to present usually in colorectal carcinomas, we prepared the ASPP2-rAd and study with L-OHP-treated HCT116 p53^−/−^ colon cancer cells. Cells were infected with GFP-rAd, ASPP2-rAd, p53-rAd and ASPP2-rAd plus p53-rAd respectively, then treated with L-OHP (100 μM) for 12 hr. Using PI staining to detect late apoptosis, we found that p53-rAd and ASPP2-rAd solely infected cells showed higher rate of cell death than control GFP-rAd-infected cells (Fig.1A a–d). The statistic graphs with 200 cells counted in three independent wells are shown in Figure1B. It showed that sole overexpression of p53 or ASPP2 and overexpression of both p53 and ASPP2 together can significantly increase the cell death induced by L-OHP (37.59%, 35.01%, and 48% *versus* 11.29%, *P *< 0.01; Fig.1B). We also compared the percentage of Annexin V^+^/GFP^+^ cells using flow cytometric analysis and found that the percentage of apoptosis in p53-rAd and ASPP2-rAd solely infected cells was significantly higher than that in control (16.1% and 17.9% *versus* 9.6%, *P* < 0.01; Fig.1C). Cells cotransfected with p53-rAd and ASPP2-rAd had the highest PI-staining and Annexin V-staining rates (26.96%, Fig.1C). To identify whether ASPP2 can stimulate the expression of pro-apoptotic genes, the total RNAs were extracted from HCT116 p53^−/−^ cells treated with GFP/ASPP2/p53-rAd infection plus L-OHP. The mRNA levels of apoptosis-related genes Bax and Bcl-2 were measured by qRT-PCR. The results showed that a 1.5-fold increase in Bax mRNA and 2.2-fold decrease in Bcl-2 mRNA were found in ASPP2-rAd-treated cells compared with GFP-rAd-treated cells (Fig.1D). Compared with GFP-rAd-treated cells, an approximately three- or fourfold increase in Bax mRNA was observed in p53 solely transfected and p53/ASPP2 cotransfected cells; however, a little increase in Bcl-2 mRNA was detected in p53 solely infected and p53/ASPP2 co-infected cells (Fig.1D). These findings suggest that p53 has a strong induction on Bax expression, and ASPP2 strongly reduced Bcl-2 expression. Results from immunoblot analysis also supported that ASPP2 reduced Bcl-2 expression and promoted the activation of caspase-3 when p53 was absent (Fig.1E). However, these results also supported that p53 is the dominant apoptosis promoter and has strong pro-apoptotic function, especially along with ASPP2.

### ASPP2 inhibits L-OHP-induced cell autophagy in a p53-independent manner

**Fig 2:**
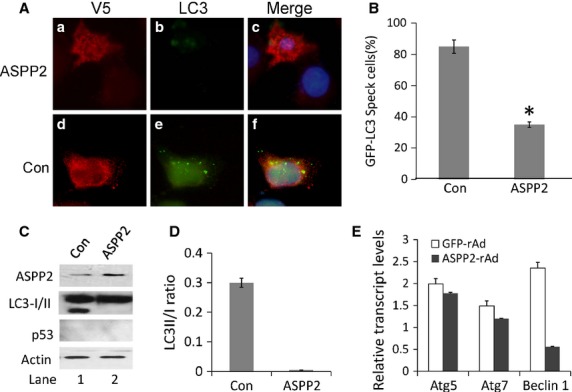
ASPP2 inhibited autophagy in a p53-independent manner. (A) Confocal microscopy of HCT116 cells transfected by the V5-ASPP2/V5-control and GFP-LC3 plasmid. Cells were stained with anti-V5 antibody (red) 48 hr after plasmid transfection. (B) Percentage of cells with GFP-LC3 puncta. Bars were cells transfected with Control and ASPP2 plasmid, respectively. Asterisks indicate statistical significance (*P* < 0.01). (C) Immunoblot analysis of cells transfected with Control and ASPP2 plasmid followed by L-OHP. Lane 1 and lane 2 were HCT116 cells transfected with control and V5-ASPP2 plasmid, respectively. (D) Bar graph of LC3II/LC3I ratio from HCT116 cells transfected with control and ASPP2 plasmid. (E) qRT-PCR analysis of Atg5, Atg7 and Beclin 1 mRNA levels of HCT116 cells-treated GFP and ASPP2-rAd; relative transcript levels of target genes were normalized with GAPDH mRNA levels.

Recent study showed that ASPP2 can inhibit autophagy through p53-independent pathway 15. In this study, we identified whether ASPP2 can affect autophagy, and its association with cell death in HCT116 p53^−/−^ cells. First, HCT116 P53^−/−^ cells were cotransfected with ASPP2/control plasmid and GFP-LC3 plasmid, followed by L-OHP treatment for 8 hr to identify the effect of ASPP2 on autophagy. The results showed that ASPP2 overexpression stained with V5-tag antibody (Fig.2A a) had a few GFP punctuate bodies (Fig.2A b) compared to control (Fig.2A d,e). When the cells were counted, over 80% of control plasmid-transfected cells were positive for GFP-LC3 specks (>8 punctuate bodies/cell), and only approximately 35% of V5-ASPP2-transfected cells contained GFP-LC3 specks (Fig.2B). These results suggested that ASPP2 inhibited L-OHP-induced cell autophagy independent of p53.

To further confirm these results, we performed Western blot to analyse the association of ASPP2 overexpression with LC3-II/LC3-I ratio in L-OHP-treated HCT116 p53^−/−^ cells. Cells were infected with ASPP2-rAd or GFP-rAd for 24 hr following L-OHP treatment for 8 hr. The results showed that LC3-II/LC3-I ratio was significantly higher in GFP-rAd-treated cells than in ASPP2-rAd-treated cells (Fig.2C). The statistic graph regarding Western blotting data for LC3-II/LC3-I ratio is shown in Figure2D. Previous study showed that ASPP2 competing with ATG16 to bind ATG5/ATG12 and preventing ATG16/ATG5/ATG12 formation. Therefore, the mRNA levels of autophagy-related genes (ATG5, ATG7 and Beclin-1) were further investigated with qRT-PCR. The results showed that ATG5 and ATG7 expression at mRNA level were not significantly different between ASPP2 overexpression and control. However, the Beclin-1 mRNA was significantly reduced in ASPP2 overexpression groups compared with control group (Fig.2E).

### ASPP2 inhibits autophagy and facilitates cell apoptosis in a p53-independent manner

**Fig 3:**
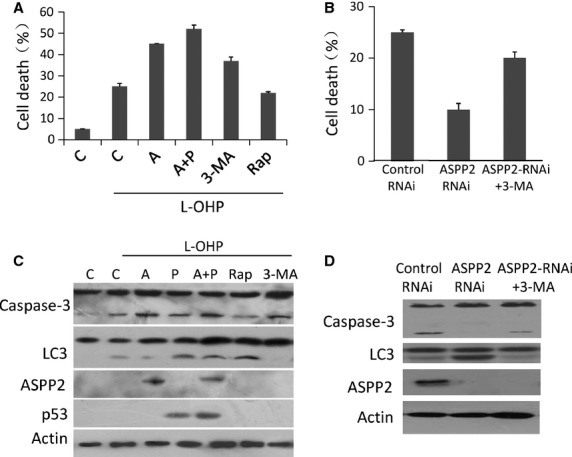
ASPP2 inhibited autophagy and facilitated apoptosis induced by L-OHP. HCT116 cells were transfected with GFP-rAd/ASPP2-GFP-rAd/p53-GFP-rAd. 3-MA or rapamycin were added to cells before treatment with L-OHP. (A) The percentage of Annexin V+/PI+-stained cells analysed by flow cytometry: Bar 1 was HCT116 cells transfected with GFP-rAd without treatment with L-OHP; Bar 2, 3, 4, 5, and 6 were all treated with L-OHP, from left to right were cells transfected with GFP-rAd, ASPP2-rAd, p53 plus ASPP2-rAd, 3-MA and rapamycin, respectively. (B) The percentage of Annexin V+/PI+-stained cells analysed by flow cytometry: Bars were Control RNAi, ASPP2 RNAi and ASPP2 RNAi plus 3-MA-treated cells from left to right, respectively. (C) Immunoblot analysis of HCT116 cells from treated cells. Lane 1 was control cells transfected with GFP-rAd without treatment with L-OHP; Lane 2, 3, 4 and 5 were GFP-rAd, ASPP2-GFP-rAd transfected cells, rapamycin and 3-MA-treated cells followed by L-OHP. (D) The percentage of Annexin V+/PI+-stained cells treated with RNAi analysed by flow cytometry; (E) Immunoblot analysis of HCT126 cells treated with RNA interference followed by L-OHP. Lane 1 was cells treated with control RNAi; Lane 2 and 3 were cells treated with ASPP2 RNAi and ASPP2-RNAi with 3-MA respectively.

The previous experiment showed that ASPP2 significantly reduced the cellular autophagy in L-OHP-treated HCT116 p53^−/−^ cells. L-OHP is a useful medicine in tumour chemotherapy, so we wonder whether the reduced autophagy by ASPP2 is associated with cell death especially in p53 inactivation situation, which is common in clinical colon cancer. To investigate this association, we performed flow cytometry assay with Annexin V/PI staining. The results showed that Annexin V and PI double-positive cells were 5% in control and up to 25% in L-OHP treatment alone. Interestingly, the cell death rates (Annexin V and PI double positive) were respectively increased to 45%, 52% and 37% in L-OHP treatment plus ASPP2-rAd, ASPP2-rAd/p53-rAd, and 3-MA groups, respectively (Fig.3A). It suggested that overexpression of ASPP2 in HCT116 P53^−/−^ cells or treatment with autophagy inhibitor (3-MA) can induce more L-OHP-treated cells into apoptosis. We also used Atg-5 siRNA-treated HepG2 cells to test our results. And we found the similar results. The results were shown in supplementary data (Fig. S2). Meanwhile, we did not find a significant difference in cell death rate between HCT116 p53^−/−^ cells treated with L-OHP alone (25%) and those treated with L-OHP plus autophagy inducer rapamycin (22%). We then knocked down ASPP2 expression with ASPP2 siRNA and reduced autophagy level with 3-MA to identify whether cell death induced by ASPP2 is related to autophagic reduction. The results showed that the cell death rate was 10% in ASPP2 RNAi-treated cells and 25% in control RNAi-treated cells. However, the cell death rate was up to 20% in ASPP2 RNAi plus 3-MA-treated cells (Fig.3B). This result supported that ASPP2-induced cell death in p53 null cells was associated with autophagic reduction. Subsequently, caspase-3 cleavage and LC3-II/LC3-1 ratio were detected by immunoblotting to confirm that ASPP2 overexpression was associated with autophagy and apoptosis. The results showed that overexpression of ASPP2 increased the level of caspase-3 17 kd fragment and decreased the LC3-II/LC3-1 ratio in L-OHP treatment groups (Fig.3C lane 3 *versus* lane 2). Meanwhile, 3-MA treatment can also reduce the LC3-II/LC3-1 ratio and induce the caspase-3 cleavage in L-OHP treatment groups (Fig.3C lane 5 *versus* lane 2). However, rapamycin and L-OHP-treated group only showed a dramatic increase in LC3-II/LC3-1 ratio, but failed to affect caspase-3 cleavage (Fig.3C lane 4 *versus* lane 2). This Western blot result supported our finding in Figure2A that increased cell death by ASPP2 overexpression might be associated with autophagic decrease in ASPP2 overexpression cells. To further confirm this finding, we ran Western blot assay with ASPP2 RNAi and ASPP2 RNAi/3-MA-treated cells. The results showed that caspase-3 cleavage was significantly reduced, and LC3-II/LC3-1 ratio was significantly increased in ASPP2 RNAi plus L-OHP-treated cells (Fig.3D lane 2 *versus* lane 1). However, the caspase-3 cleavage was not significantly different between control RNAi-treated group and ASPP2 RNAi/3-MA-treated group (Fig.3D lane 3 *versus* lane 1), while LC3-II/LC3-1 ratio was significantly reduced by 3-MA treatment in ASPP2 RNAi/3-MA-treated group (Fig.3D lane 3 second raw). These results provided lateral evidence suggesting that ASPP2 inhibited autophagy and facilitated cell apoptosis. When the cell autophagy function was restored, cell apoptosis induced by ASPP2 was decreased.

### Mouse model confirmed that ASPP2 was involved in the reduction of autophagy in colon tissue

**Fig 4:**
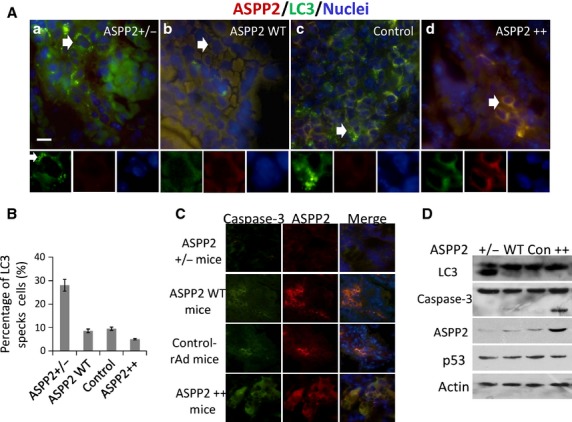
Autophagy and apoptosis in colon tissues from L-OHP-treated transgenic mice. Mice overexpressing ASPP2 were made from ASPP2 WT Balb/c mice by injecting ASPP2-rAd through tail vein. Control mice were ASPP2 WT injected with Control-rAd. Accompanied by 10 days administration, mice were injected with L-OHP through tail vein, simultaneously. (A) Double IF staining of transgenic mice colon section with anti-ASPP2 (red) and anti-LC3 (green) antibodies. a, b, c and d were ASPP2^+/−^, ASPP2 WT, Control and ASPP2 overexpression mice respectively. (B) Percentage of LC3 specks cells. (C) Double IF staining of transgenic mice colon section with anti-ASPP2 (red) and anti-active caspase-3 (green) antibodies. (D) Immunoblot analysis of transgenic mice colon tissues. Lane 1, 2, 3 and 4 were ASPP2^+/−^ mice, ASPP2 WT, Control and ASPP2 overexpression mice, respectively.

To identify the effect of ASPP2 on autophagy in the colon tissue of L-OHP-treated mice, we detected LC3 specks with immunofluorescence and LC3-II/LC3-I ratio with Western blot in ASPP2 low expression (ASPP2^+/−^) mice 16 and ASPP2 overexpressing mice obtained by injecting ASPP2-rAd to ASPP2 WT mice through tail vein for 24 hr. All the mice were treated with L-OHP through intraperitoneal injection for 48 hr. Colon sections were made from freshly prepared colon tissues and double-stained with anti-ASPP2 and anti-LC3 antibodies. LC3 specks were observed in most of the cells from ASPP2^+/−^ mice, but only a few were detected in ASPP2 WT mice (Fig.4A a,b). Then, we detected LC3 specks in ASPP2-rAd-treated mice and control-rAd-treated mice. The results showed that LC3 specks were not observed in ASPP2-rAd-infected colon cells, and a few were detected in control-rAd-infected colon cells (Fig.4A c,d). We counted 100 LC3-positive cells, calculated the percentage of cells with LC3 specks, and performed statistical analysis with three repeated counting. The results showed that the percentage of LC3 specks-positive cells was much higher in ASPP2^+/−^ mice, compared with ASPP2 WT and GFP-rAd- and ASPP2-rAd-injected mice (*P* < 0.05; Fig.4B). We further detected caspases-3 expression *in situ* with immunofluorescence using mouse colon tissues. It was found that in ASPP2-overexpressing mouse colon tissues, caspases-3 levels increased. But in ASPP2^+/−^, WT and control mice, caspase-3 was seldom detected, especially in ASPP2^+/−^ mice (Fig.4B). This result suggested that ASPP2 overexpression might have enhanced the apoptosis induced by L-OHP. The same result was confirmed by Western blot analysis, *i.e*. significant amounts of LC3-II were detected in ASPP2^+/−^ mice, but very few in ASPP2 WT mice (Fig.4C). Likewise, in ASPP2 overexpression mice, active caspase-3 was detected (Fig.4C lane 4). These results further confirmed that ASPP2 inhibited L-OHP-induced autophagy and improved the efficacy of L-OHP.

### The ASPP2 mRNA expression was significantly lower and the ratio of p53 mutation was higher in colon tumour tissues than in corresponding adjacent non-tumour tissues

p53 mutations and inactivation of p53 apoptotic pathway are found in almost 50% of human cancers 17. ASPP2 is reported to be a tumour suppressor and closely associated with cell apoptosis 18. Above results suggested that ASPP2-reduced autophagy contributed to cell death in a p53-independent manner. In this study, we detected p53 mutation and ASPP2 expression in 20 pairs of colon cancer samples and their adjacent tissues.

**Fig 5:**
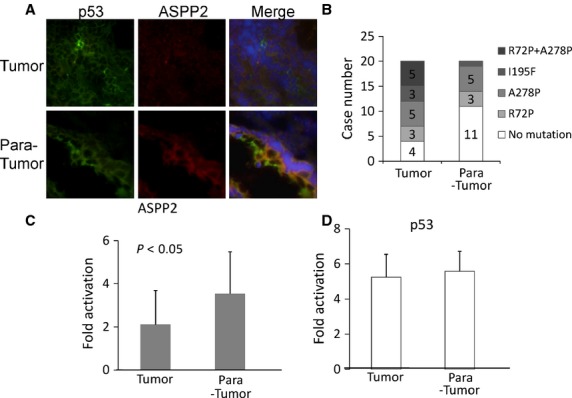
ASPP2 mRNA expressed lower in tumour tissues of colorectal cancer than in adjacent normal tissues. (A) Double IF staining of human colon cancer and para-tumour tissues using anti-ASPP2 (red) and p53 (green) antibodies; (B) Case numbers of p53 transcript mutations in tumour and para-tumour tissues; (C) qRT-PCR analysis of ASPP2 mRNA levels in 20 colorectal cancer tissues and their para-tumour tissues; relative transcript levels of target genes were normalized with GAPDH mRNA levels; (D) qRT-PCR analysis of p53 mRNA levels in 20 colorectal cancer tissues and their para-tumour tissues, relative transcript levels of target genes were normalized with GAPDH mRNA levels.

ASPP2 can significantly increase p53-mediated cell death 18. Our *in vitro* results supported that ASPP2 can stimulate colon cancer cell death in a p53-independent manner. The expression of ASPP2 is frequently suppressed in many human cancers, such as breast cancer 19 and lymphoma subtypes 11. Mouse models targeting ASPP2 demonstrated that ASPP2 is a haploinsufficient tumour suppressor 16. By using frozen section of colon cancer, we first double-stained p53 and ASPP2 and showed that ASPP2 expression was higher in adjacent normal tissues than in tumour tissues, but no differences in p53 expression were found between tumour and adjacent normal tissues (Fig.5A). To identify p53 mutations, we prepared total RNA from these frozen tumour and adjacent tissues and ran RT-PCR assay. By direct sequencing of PCR products, three unique mutation sites were found, *i.e*. R72P, A278P and I195F, in 16 tumour tissues and 9 adjacent tissues. Most of them were single mutation, only five colon cancer tissues were R72P and A278P double mutations, and no triple mutations were detected in the samples (Fig.5B). More mutations were found in tumour tissues than in adjacent tissues, and nine samples were found having the same p53 mutation in both adjacent and tumour tissues. These patients would be the high-risk population for colon cancer because of the p53 mutations. Our result also supported previous studies that more than 50% colon cancers display inactivated p53.

A total of 20 colon cancer tissues and their corresponding adjacent non-neoplastic tissues were used to assess the ASPP2 and p53 mRNA levels with qRT-PCR. ASPP2 mRNA was found significantly lower in tumour tissues than in adjacent tissues (Fig.5C, *P* < 0.05), and the fold changes of ASPP2 mRNA expression varied from 0.28 to 8.37. The p53 mRNA levels in all these specimens were determined, which failed to show significant difference between tumour and adjacent tissues (Fig.5D). These results further suggested that ASPP2 expression was independent of p53, and its low level in colon cancer would play a negative role in chemotherapy of colon cancer. Considering the p53 mutation results, our data supported that ASPP2 expression was higher in adjacent normal tissues than in tumour tissues and independent of either p53 mutation or expression level.

## Discussion

Inactivation of cell death pathways is a central component of cancer progression 20. The ability of a tumour cell to evade programmed cell death (apoptosis) and multiply is crucial in the development of cancer 21. p53 is one of the mostly studied tumour suppressors, and over 50% human tumours carry p53 mutations 17,22. Mutant forms of p53 lack their pro-apoptotic function and decrease the sensitivity of tumour cells to anticancer drugs 23–25. Abnormal p53 was associated with increased risk of death and failure of response to radiotherapy in patients with colorectal cancer 26. Novel strategies and molecules which can convert the abnormal conformations of mutant p53 to normal p53 or enhance the apoptosis of tumour cells will be attractive therapeutic targets for cancer treatment 27.

Among the p53 interacting proteins, ASPP2 is one that specially stimulates the pro-apoptotic function of p53 and is regarded as a tumour suppressor. Mori *et al*. found that cell lines expressing high levels of ASPP2 mRNA were more sensitive to UV, X-ray irradiation and chemotherapy drug CDDP (cis-diamine-dichloroplatinum), whereas those expressing low levels were comparatively resistant 28. In this study, ASPP2 was found to increase apoptosis induced by L-OHP in a p53-independent manner through inhibiting autophagy. When ASPP2 was silenced, cell autophagy increased, and apoptosis decreased. This phenomenon indicated that reduced ASPP2 in cells created conditions for tumour formation. It reduced damaged cell death and accumulated injuries in cells, which was beneficial for tumour survival. On the other hand, ASPP2 enhanced cell apoptosis under chemotherapy, which sensitized the tumour to chemotherapy. Loss of ASPP2 contributed to the significant decrease in sensitivity to anticancer drugs in tumour cells. Therefore, ASPP2 could be hopefully a therapeutic target for cancer treatment. However, it must be pointed out that ASPP2 overexpression could lead to the apoptosis of normal cells in L-OHP-treated mice (Fig.4B and C). Therefore, too much ASPP2 is also an egregious element. Keeping ASPP2 at a normal expression level will be crucial to avoid tumour formation.

The precise molecular mechanisms behind ASPP2 promoting cell apoptosis remain to be elucidated. In this study, we found that ASPP2 enhanced cell apoptosis through inhibiting cell autophagy induced by L-OHP. When cells were treated with autophagy inhibitor 3-MA, the apoptosis reduced by ASPP2 knockdown could be reversed. It confirmed that reduction in autophagy through ASPP2 contributed to the cell death induced by L-OHP. Autophagy is an intracellular degradative pathway that targets cytosolic components to lysosomes to be degraded for the purposes of maintaining cellular homoeostasis and supplying substrates for energy generation 29. During apoptosis, the stimulation of autophagy can be either a protective mechanism or a process that contributes to cell death 30. The currently more-accepted conclusion is that in response to different stimuli, autophagy may act to selectively target different protein or organelle cargos 31. Our finding demonstrated that ASPP2 at least induces cell apoptosis by inhibiting autophagy pathway, though the precise molecular mechanism is still unclear. ASPP2 was reported to inhibit autophagy through interaction with ATG5 18, and ASPP2 can be located in mitochondria 32. All these findings provide clues for mechanism investigation.

Down-regulation of ASPP2 was observed in human breast tumours 19 and human lung cancer cell lines 28. ASPP2 levels were also found to be linked with poor prognosis and metastasis in human cancers 11,12. In this study, we found that more than half (16/20) of the colorectal cancer tissues carried p53 mutations, especially in tumour tissues. ASPP2 mRNA expression was higher in adjacent normal tissues than tumour tissues and independent of both p53 mutation and expression level. The unbalanced expression of ASPP2 and p53 in colorectal cancers also suggested that ASPP2 might act as a tumour suppressor solely independent of p53. Nevertheless, only 20 patients with cross-sectional data were investigated in this study, and varied ASPP2 expression might result in different disease outcome.

Taken together, this study revealed a novel function of ASPP2 in modulating autophagy and apoptosis. Low levels of ASPP2 in colorectal tumours would contribute to the tumour survival in both p53-dependent and -independent manners following chemotherapy. It may shed light on the understanding of tumour suppression and lead to the development of novel agents for cancer therapy.

## Supporting Information

**Figure S1** Percentage of Annexin V+/PI cells under different concentration and different time period of L-OHP treatment

**Figure S2** ASPP2 inhibited autophagy and enhanced apoptosis in Atg5 siRNA treated HCT116 (p53^−/−^) cells.
