# Supplementary figures and images for: ASPP2 enhances Oxaliplatin (L-OHP)-induced colorectal cancer cell apoptosis in a p53-independent manner by inhibiting cell autophagy
Source: J Cell Mol Med. 2014 Dec 23;19(3):535–43. doi: 10.1111/jcmm.12435 (PMC4369811; doi:10.1111/jcmm.12435)

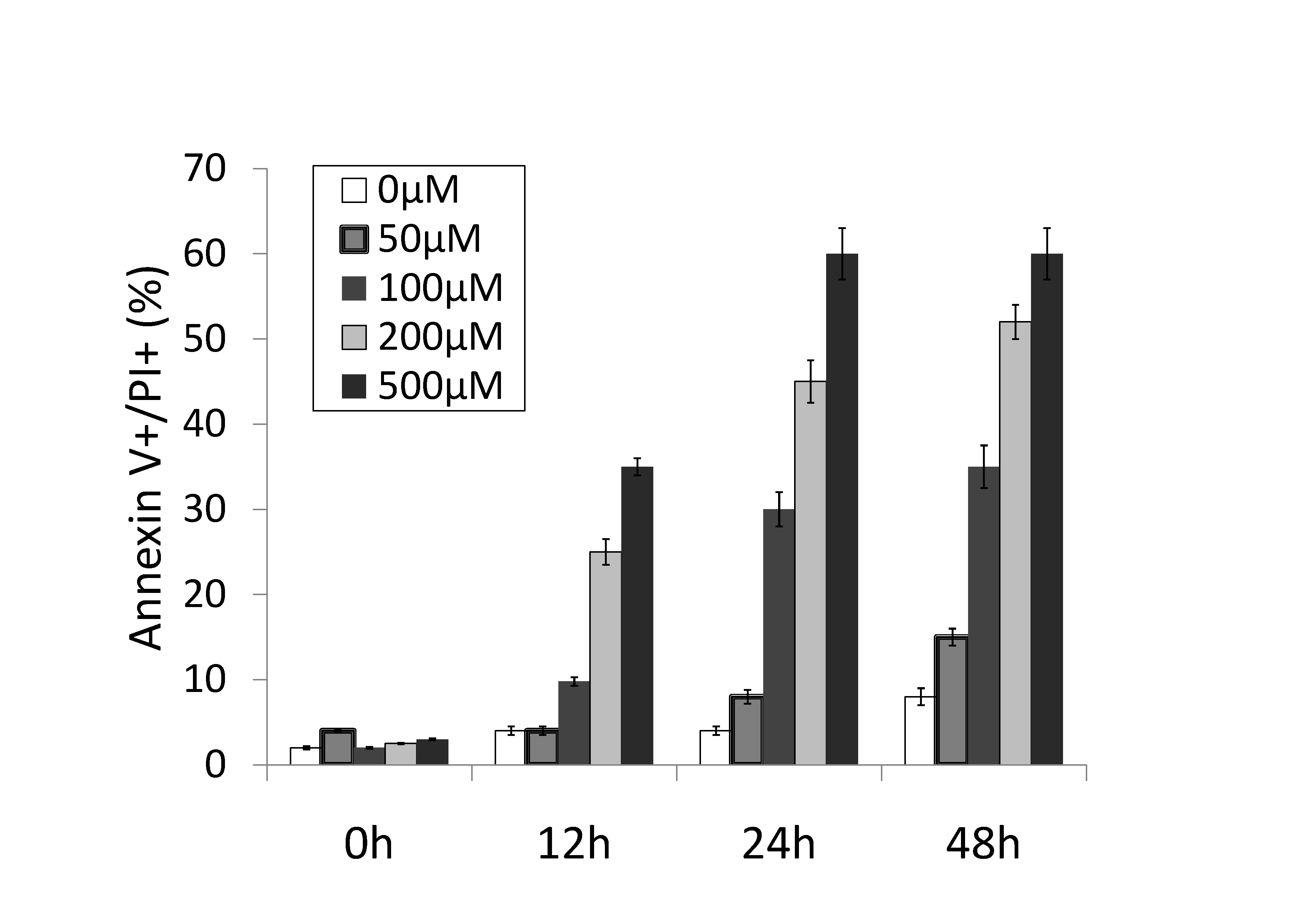

Supplement: Supplementary file 1 [file jcmm0019-0535-sd1.tif]

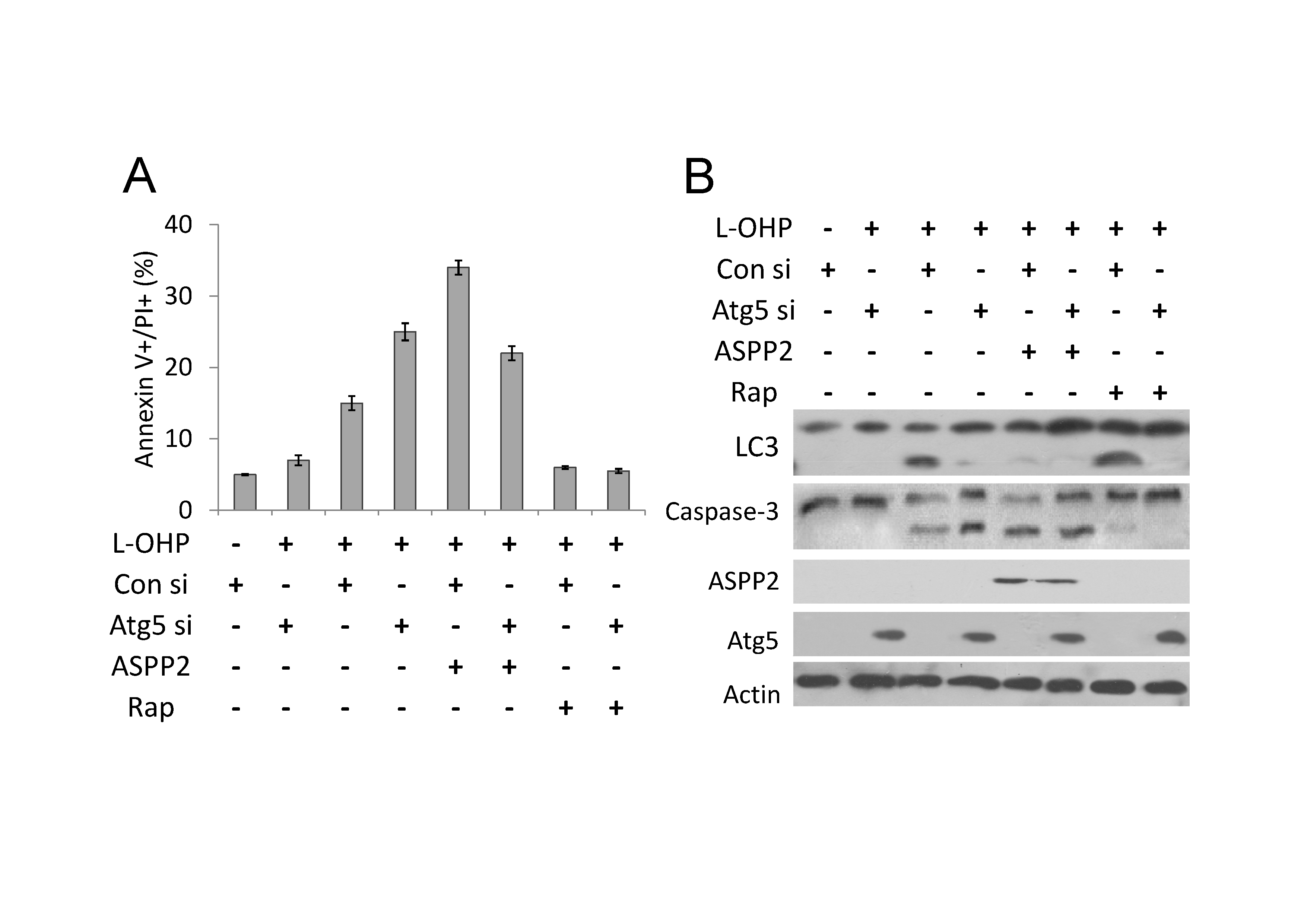

Supplement: Supplementary file 2 [file jcmm0019-0535-sd2.tif]
