# Supplementary material for: ASPP2 enhances Oxaliplatin (L-OHP)-induced colorectal cancer cell apoptosis in a p53-independent manner by inhibiting cell autophagy
Source: J Cell Mol Med. 2014 Dec 23;19(3):535–43. doi: 10.1111/jcmm.12435 (PMC4369811; doi:10.1111/jcmm.12435)
Supplement: Supplementary file 3 [file jcmm0019-0535-sd3.docx]

Supporting Information:

Fig S1: Percentage of Annexin V+/PI cells under different concentration and different time period of L-OHP treatment.

Fig S2: ASPP2 inhibited autophagy and enhanced apoptosis in Atg5 siRNA treated HCT116 (p53-/-) cells.
